# Correction: Acute inhibition of neurosteroid estrogen synthesis suppresses status epilepticus in an animal model

**DOI:** 10.7554/eLife.19109

**Published:** 2016-07-01

**Authors:** Satoru M Sato, Catherine S Woolley

Sato SM, Woolley CS. 2016. Acute inhibition of neurosteroid estrogen synthesis suppresses status epilepticus in an animal model. *eLife* **5**:e12917. doi: 10.7554/eLife.12917.

Published April 15, 2016

We identified an error in the y-axis scale of Figure 2G, which was mislabeled 0 to 100% in the published article and should have been labeled 0 to 80%. Correcting this error does not affect any of the quantitative data reported in the text or the conclusions of the study.

The corrected Figure 2 is shown here:

**Figure 1:**
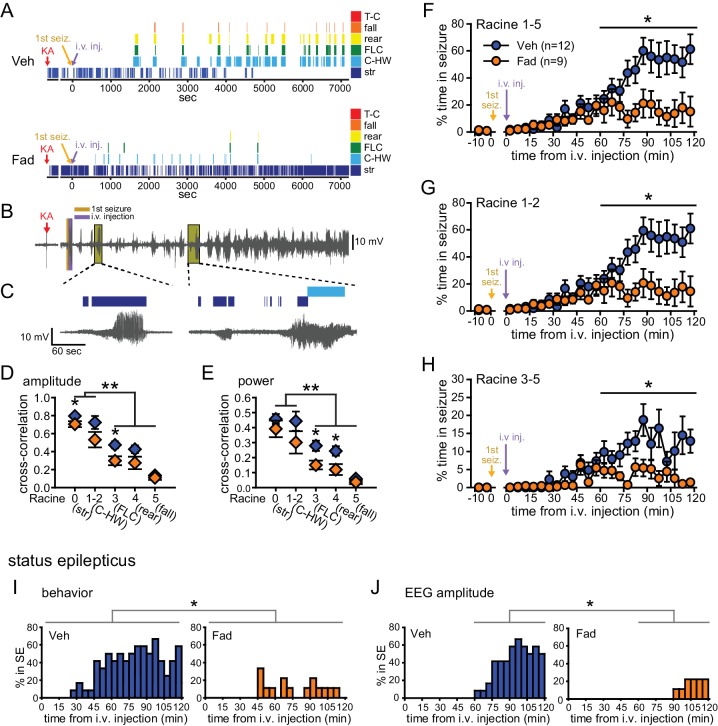

The originally published Figure 2 is also shown for reference:

**Figure 2:**
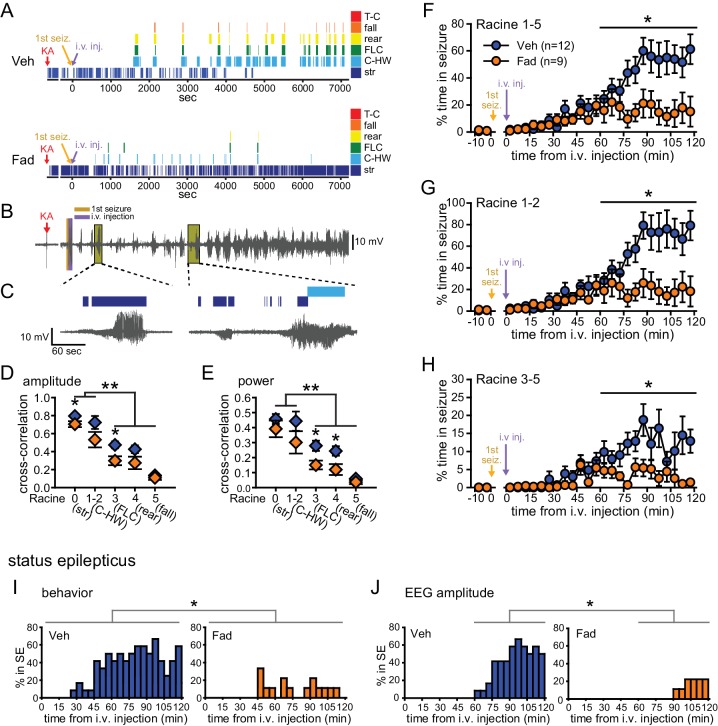

The article has been corrected accordingly.

