# Supplementary material for: Acute inhibition of neurosteroid estrogen synthesis suppresses status epilepticus in an animal model
Source: eLife. 2016 Apr 15;5:e12917. doi: 10.7554/eLife.12917 (PMC4862752; doi:10.7554/eLife.12917)
Supplement: Supplementary file 1. — Each table lists significant effects shown in each Figure with the respective mean sample size and Cohen’s d statistic for each effect. DOI: http://dx.doi.org/10.7554/eLife.12917.021 [file elife-12917-supp1.docx]

**Supplementary File 1 – Summary of Effect Sizes**

| **Systemic fadrozole on EEG** | | | | |
| --- | --- | --- | --- | --- |
| *figure* | *panel* | *comparison* | *mean sample size* | *Cohen’s d* |
| Figure 1 | D | Veh - Fad (2^nd^ hr) | 10.5 | 1.50 |
|  | E | Veh - Fad (2^nd^ hr) | 10.5 | 2.04 |
|  | G | Veh - Fad (2^nd^ hr) | 10.5 | 1.62 |
|  | H | Veh - Fad (2^nd^ hr) | 10.5 | 1.11 |
|  |  |  |  |  |
| **Systemic fadrozole on behavioral seizures** | | | | |
| *figure* | *panel* | *comparison* | *mean sample size* | *Cohen’s d* |
| Figure 2 | D | str - FLC | 20.5 | 2.72 |
|  | D | str - rear | 20.0 | 2.76 |
|  | D | str - fall | 15.5 | 7.03 |
|  | D | C-HW - FLC | 20.5 | 1.12 |
|  | D | C-HW - rear | 20.0 | 1.22 |
|  | D | C-HW - fall | 15.5 | 2.32 |
|  | D | Veh - Fad (str) | 10.5 | 1.00 |
|  | D | Veh - Fad (FLC) | 9.5 | 1.30 |
|  | E | str - FLC | 20.5 | 1.59 |
|  | E | str - rear | 20.0 | 1.81 |
|  | E | str - fall | 15.5 | 3.21 |
|  | E | C-HW - FLC | 20.5 | 0.88 |
|  | E | C-HW - rear | 20.0 | 1.03 |
|  | E | C-HW - fall | 15.5 | 1.75 |
|  | E | Veh - Fad (FLC) | 10.0 | 1.26 |
|  | E | Veh - Fad (rear) | 9.5 | 1.20 |
|  | F | Veh - Fad (2^nd^ hr) | 10.5 | 1.09 |
|  | G | Veh - Fad (2^nd^ hr) | 10.5 | 1.10 |
|  | H | Veh - Fad (2^nd^ hr) | 10.5 | 1.18 |
|  | I | Veh - Fad | 10.5 | 1.44 |
|  | J | Veh - Fad (2^nd^ hr) | 10.5 | 1.69 |
|  |  |  |  |  |
| **4-dione retrodialysis** | | | | |
| *figure* | *panel* | *comparison* | *mean sample size* | *Cohen’s d* |
| Figure 3 | E | pre - post 4-dione retrodialysis | 10.0 | 1.41 |
|  |  |  |  |  |
| **E2 microdialysis during seizures** | | | | |
| *figure* | *panel* | *comparison* | *mean sample size* | *Cohen’s d* |
| Figure 4 | A | BL - post-KA (1^st^ hr) | 31.0 | 0.76 |
|  | A | BL - post-KA (2^nd^ hr) | 31.0 | 0.93 |
|  | B | BL - post-KA (2^nd^ hr) | 10.0 | 1.07 |
|  | C | BL - post-KA (1^st^ hr) | 17.0 | 1.08 |
|  | C | BL - post-KA (2^nd^ hr) | 17.0 | 1.04 |
|  | E | BL (mild/mod) - BL (severe) | 15.5 | 1.19 |
|  | E | BL (mild/mod) - post-KA (mild/mod) | 27.0 | 0.69 |

| **Intra-hippocampal fadrozole on EEG** | | | | |
| --- | --- | --- | --- | --- |
| *figure* | *panel* | *comparison* | *mean sample size* | *Cohen’s d* |
| Figure 5 | C | str - FLC | 26.00 | 2.28 |
|  | C | str - rear | 25.50 | 1.97 |
|  | C | str - fall | 20.50 | 3.68 |
|  | C | C-HW - FLC | 20.00 | 2.59 |
|  | C | C-HW - rear | 19.50 | 2.12 |
|  | C | C-HW - fall | 14.50 | 4.42 |
|  | D | str - FLC | 26.00 | 1.61 |
|  | D | str - rear | 25.50 | 1.38 |
|  | D | str - fall | 20.50 | 2.45 |
|  | D | C-HW - FLC | 20.00 | 1.77 |
|  | D | C-HW - rear | 19.50 | 1.47 |
|  | D | C-HW - fall | 14.50 | 2.67 |
|  | F | Veh - Fad | 10.50 | 1.06 |
|  | H | Veh - Fad | 10.50 | 1.15 |
|  |  |  |  |  |
| **Intra-hippocampal fadrozole on behavioral seizures** | | | | |
| *figure* | *panel* | *comparison* | *mean sample size* | *Cohen’s d* |
| Figure 6 | A | Veh - Fad | 29.00 | 0.70 |
|  | C | Veh - Fad (2^nd^ hr) | 16.00 | 1.13 |
|  | D | Veh - Fad (2^nd^ hr) | 16.00 | 1.00 |
| **Systemic letrozole on EEG** | | | | |
| *figure* | *panel* | *comparison* | *mean sample size* | *Cohen’s d* |
| Figure 7 | D | Veh - Let | 10.50 | 0.81 |
|  | E | Veh - Let | 10.50 | 0.76 |
|  | H | Veh-Let (δ-θ) | 10.50 | 0.77 |
|  | H | Veh-Let (β-low γ) | 10.50 | 0.72 |

| **Systemic letrozole on status epilepticus** | | | | |
| --- | --- | --- | --- | --- |
| *figure* | *panel* | *comparison* | *mean sample size* | *Cohen’s d* |
| Figure 8 | C | Veh - Let | 10.50 | 0.76 |
|  | D | Veh - Let | 10.50 | 0.99 |
